# A Digital Model to Explain the Necessity of Prostaglandin E1 After Balloon Atrial Septostomy in D-Transposition of the Great Arteries

**DOI:** 10.3390/jcdd13080370

**Published:** 2026-08-04

**Authors:** Fabio Savorgnan, Saul Flores, Julia Garcia-Mancebo, Adel Hassan, Rohit S. Loomba, Sebastian Acosta

**Affiliations:** 1 Department of Pediatrics, Division of Critical Care, Baylor College of Medicine and Texas Children’s Hospital, Houston, TX 77030, USA; saul.flores2@bcm.edu (S.F.);; 2 Department of Medicine, University of Texas Southwestern, Dallas, TX 75390, USA; 3 Department of Pediatrics, Division of Cardiology, Northwestern University, Evanston, IL 60611, USA; rohit.loomba@northwestern.edu

**Keywords:** Transposition of the Great Vasel (TGA), Ballon Atrial Septostomy (BAS), computational model

## Abstract

Objective: Patients with D-transposition of the great arteries (TGA) often require prostaglandin E1 (PGE) even after balloon atrial septostomy (BAS). This study builds a computer simulation that analyzes whether or not elevated pulmonary vascular resistance (PVR) could explain the profound hypoxemia seen in some patients after PGE discontinuation. Methods: We developed a systems-based mathematical model of TGA incorporating systemic and pulmonary circulations, an atrial septal defect (ASD), and a patent ductus arteriosus (PDA), with bidirectional atrial mixing. The PGE-on pre-BAS state represented restrictive atrial communication, with systemic arterial saturation in the clinically expected 60–70% range. BAS was modeled by reducing ASD resistance and increasing the atrial mixing parameter; PGE withdrawal was modeled by increasing PDA resistance. A reproducible Monte Carlo cohort of 500 virtual patients was generated using independent probability distributions for heart rate, PVR, SVR, ASD resistance, PDA resistance, and atrial mixing. Prespecified sensitivity analyses varied ASD resistance reduction, PDA resistance, and pulmonary and systemic vascular responses to PGE withdrawal. Results: The PGE-on pre-BAS cohort had a median systemic arterial saturation of 64.7% (interquartile range, 59.1–68.8%), which increased to 74.7% (70.6–77.9%) after BAS. Following PGE withdrawal, saturation decreased to 70.9% (66.0–74.4%), while systemic flow increased from 1.83 to 2.04 L/min/m^2^ and systemic oxygen delivery increased from 271.4 to 283.9 mL O_2_/min/m^2^. For the response from PGE-on post-BAS to PGE-off post-BAS, each 1-WU·m^2^ increase in PVR was associated with a 0.56-percentage-point greater decrease in saturation, a 6.56% smaller increase in systemic flow, a 3.96% greater decrease in effective pulmonary flow, and a 7.87% smaller increase in oxygen delivery (all *p* < 0.01). Conclusions: In a model representing clinically restrictive pre-BAS atrial communication, PVR strongly modified the response to PGE withdrawal after BAS. Higher PVR was associated with larger decreases in saturation and effective pulmonary flow and smaller improvements in systemic flow and oxygen delivery. Saturation and oxygen delivery may move in opposite directions; therefore, assessment after PGE withdrawal should integrate systemic perfusion rather than rely on saturation alone. These findings are mechanistic and require external clinical validation.

## 1. Introduction

In D-transposition of the great arteries (TGA), a mixing lesion is necessary to shunt oxygen from the pulmonary circulation to the systemic circulation. The three potential mixing lesions are an atrial septal defect (ASD), ventricular septal defect (VSD), and a patent ductus arteriosus (PDA) [1]. However, if mixing is insufficient, it can be increased at the ASD by balloon atrial septostomy (BAS) and at the PDA by prostaglandin E1 (PGE). In a previous retrospective analysis, we analyzed the effectiveness that BAS has in the successful discontinuation of PGE by increasing the mixing at the atrial level [2]. We were surprised to find that BAS was not effective enough to discontinue PGE in the majority of patients before they underwent the arterial switch operation. Other investigators have also found that approximately 20–60% of patients continue to require PGE after BAS [3,4,5]. The objective of the present study is to implement a mathematical model to explain the interaction between BAS, discontinuation of PGE (closure of PDA), and other hemodynamic parameters including pulmonary vascular resistance (PVR) and systemic vascular resistance (SVR) in order to find hemodynamic conditions that hinder the effectiveness of the BAS. In clinical practice, BAS is performed when restrictive interatrial communication causes inadequate mixing and severe hypoxemia, commonly with systemic arterial saturation below approximately 60–70% [2,3,4,5].

## 2. Materials and Methods

### 2.1. Mathematical Model

We developed a mathematical model of the circulatory system in patients with TGA. This model contains components to represent systemic circulation, pulmonary circulation, cardiac ventricles and atria, the PDA, and the ASD. This model also accounts for the intake of oxygen (O_2_) in the lungs, the transport of O_2_ in the circulation, the bidirectional flow through the ASD, and its consumption in the systemic organs. We are able to model the effect of the BAS by modifying the flow resistance through the ASD and the effect of PGE by modifying the flow resistance through the PDA. The model also allows us to simulate high/low-PVR and high/low-SVR conditions which in clinical practice can be manipulated by vasopressors or vasodilators.

Our model assumes two parallel circulations, one linking the right ventricle (RV) and systemic circulation, and the other connecting the left ventricle (LV) and pulmonary circulation. The two mixing lesions are the PDA, which allows unidirectional blood flow from systemic to pulmonary circulation, and the ASD, which allows bidirectional flow between the systemic and pulmonary circulation at the atrial level. This bidirectional mixing in the ASD is a key feature of our model which improves accuracy of oxygen saturations. It is parameterized by a variable $G_{\mathrm{asd}}$, which represents the reverse flow through the ASD as a fraction of the forward flow. If there is unidirectional forward flow from the left atrium (LA) to the right atrium (RA), $G_{\mathrm{asd}}$ becomes zero.

The oxygen saturation of the blood is influenced by two key junction points in the model, J1 at the pulmonary artery and J2 at the right atrium. At J1, deoxygenated blood from the right ventricle flows through the PDA to join with mostly oxygenated blood from the left ventricle, producing the oxygen saturation of the pulmonary artery. The blood from the left ventricle is mostly oxygenated because it consists primarily of blood from the pulmonary veins, along with some retrograde flow of deoxygenated blood from the ASD. At J2, where oxygenated blood from the pulmonary veins flows through the ASD to mix with deoxygenated blood from the systemic veins before entering the RV.

Below is a complete listing of all equations, whose roots we solved using the optimize function of the SciPy library version 1.9.1. The first equation is

(1) $$Q_{s}+Q_{pda}=HR\times \left(C_{RV,dia}\times P_{sv}-C_{RV,sys}\times P_{sa}\right)$$

which dictates that the steady cardiac output of the *RV* is the product of the heart rate (HR) and the stroke volume. Here, $Q_{s}$ and $Q_{pda}$ represent the flow through the systemic circulation and the PDA, respectively. $C_{RV,dia}$ and $C_{RV,sys}$ denote the compliances of the *RV* at diastole and systole, and $P_{sv}$ and $P_{sa}$ represent the blood pressure in the systemic veins and systemic arteries, respectively. The blood that exits the *RV* will either go into the systemic circulation or into the PDA, hence $Q_{s}+Q_{pda}$ equals the output from the *RV*. On the right-hand side, the stroke volume is equal to the end-diastolic volume (EDV) minus the end-systolic volume (ESV). In this simplified model, the diastolic pressure of the *RV* equalizes with the right atrium and by extension the central veins, hence the use of $P_{sv}$ to calculate EDV. Because of transposition, the systolic pressure in the *RV* equalizes with the systemic arteries, hence the use of $P_{sa}\;$ to calculate ESV.

The second equation of the system is

(2) $$Q_{p}-Q_{asd}=HR\times \left(C_{LV,dia}\times P_{pv}-C_{LV,sys}\times P_{pa}\right)$$

which is analogous to (Equation (1)) but for the *LV*. The main differences are that the end-diastolic and end-systolic pressures equalize with the pulmonary vein ($P_{pv}$) and the pulmonary artery ($P_{pa}$) pressures, respectively, and that the volume of blood entering the *LV* consists of flow to the pulmonary circulation ($Q_{p}$) minus the blood flow that exits through the ASD ($Q_{asd}$). Similarly to the *RV*, $C_{LV,dia}$ and $C_{LV,sys}$ denote the compliances of the *LV* at diastole and systole.

The ventricular volume terms in Equations (1) and (2) use the linear compliance-chamber relation V(t) = V0 + C(t)P(t), where V0 is the unstressed ventricular volume and C(t) is time-varying ventricular compliance. Compliance is the reciprocal of elastance and differs between end diastole and end systole: Cdia denotes the larger end-diastolic compliance and Csys the smaller end-systolic compliance. End-diastolic pressure is approximated by the corresponding atrial or venous pressure, and end-systolic pressure by the corresponding arterial pressure. Because the same V0 is present in both end-diastolic and end-systolic volume, it cancels when stroke volume is calculated as EDV minus ESV, yielding SV = Cdia × PED-Csys × PES. This formulation follows established lumped-parameter cardiovascular models using time-varying ventricular compliance [6,7].

The third equation is

(3) $$Q_{s}=\frac{P_{sa}-P_{sv}}{SVR}$$

which represents Ohm’s law of fluid flow for the systemic vasculature. Similarly, the fourth equation is

(4) $$Q_{p}=\frac{P_{pa}-P_{pv}}{PVR}$$

which represents Ohm’s law of fluid flow for the pulmonary vasculature. The analogous law for the flow through the ASD is given by a fifth equation

(5) $$Q_{asd}=\frac{P_{pv}-P_{sv}}{R_{asd}}$$

and through the PDA by a sixth equation

(6) $$Q_{pda}=\frac{P_{sa}-P_{pa}}{R_{pda}}$$

where $R_{asd}$ and $R_{pda}$ represent the resistance to flow in the ASD and PDA, respectively.

The seventh equation is

(7) $$Q_{asd}=Q_{pda}$$

which dictates that, at steady state, the flow through the ASD must equal the flow through the PDA due to conservation of blood mass.

Conservation of mass of oxygen applied at point J1 is given by an eighth equation

(8) $$Q_{p}\times S_{pa}=Q_{pda}\times S_{sa}+\left(Q_{p}-Q_{asd}\right)\times \frac{Q_{p}\times S_{pv}+G_{\mathrm{asd}}\times Q_{asd}\times S_{sv}}{Q_{p}+G_{\mathrm{asd}}\times Q_{asd}}$$

where the PDA joins with blood flow from the *LV* before flowing into the pulmonary circulation. $Q_{p}\times S_{pa}$ represents the mass of oxygen leaving point J1 toward the lungs. Here, $S_{pa}$ denotes the oxygen saturation in the pulmonary arteries. $Q_{pda}\times S_{sa}$ gives the oxygen mass entering point J1 from the PDA, since the PDA draws blood from the systemic arteries whose oxygen saturation is $S_{sa}$. The rest of the oxygen entering point J1 comes from the *LV*, which itself consists of a component from the pulmonary veins and another component from the systemic veins via the ASD. $Q_{p}-Q_{asd}$ represents the volume of blood coming into point J1 from the *LV*. This quantity is multiplied by the saturation at the LA which in turn is a mix of oxygen contents of the pulmonary and systemic veins due to the bidirectional flow at the ASD. The last equation is

(9) $$\left(Q_{s}+Q_{asd}\right)\times S_{sa}=Q_{s}\times S_{sv}+\left(1+G_{\mathrm{asd}}\right)\times Q_{asd}\times S_{pv}$$

which represents conservation of oxygen mass applied at point J2, where blood from the systemic veins joins with blood from the ASD before entering the *RV*. $Q_{s}+Q_{asd}$ represents the total volume of blood leaving point J2 to go into the *RV*. This blood flows into the systemic arteries with its oxygen saturation denoted by $S_{sa}$ and into the PDA whose flow is assumed to be unidirectional. Thus, the left-hand side of the equation is the total oxygen content flowing from the *RV*. This oxygen comes from two sources, which are represented on the right-hand side of the equation. $Q_{s}\times S_{sv}$ gives the oxygen coming into point J2 from the systemic veins whose saturation is $S_{sv}$, and the second term gives the oxygen coming into point J2 from the ASD. The volume of this blood is $\left(1+G_{\mathrm{asd}}\right)\times Q_{asd}$, and the saturation of this blood is $S_{pv}$ because the ASD allows oxygenated blood from the pulmonic vein to flow into the RA.

The next equation

(10) $$CVO2=13.4\times Hb\times Q_{s}\times \left(S_{sa}-S_{sv}\right)$$

represents Fick’s principle rearranged to solve for the rate of oxygen consumption (CVO2) by systemic tissues. Here Hb denotes the hemoglobin level. It is also useful to define an effective pulmonary flow ($Q_{p,\mathrm{eff}}$) that satisfies

(11) $$CVO2=13.4\times Hb\times Q_{p,\mathrm{eff}}\times \left(S_{pv}-S_{sv}\right)$$

from applying Fick’s principle to the pulmonary veins and the systemic veins to quantify the volume of blood which starts out deoxygenated in the systemic veins and ends up as oxygenated blood in the pulmonary vein [1]. This effective pulmonary blood flow $Q_{p,\mathrm{eff}}$ represents the portion of $Q_{p}$ that needs to be oxygenated, excluding recirculating blood that was already oxygenated.

The PGE-on pre-BAS state was defined to represent the restrictive interatrial communication for which BAS is clinically performed. The central pre-BAS ASD resistance was 7.0 WU·m^2^ and the atrial bidirectional mixing parameter G was 0.02, producing a cohort median systemic arterial saturation within the clinically expected 60–70% range. The primary BAS state was represented by a 50% reduction in ASD resistance and multiplication of the bidirectional mixing parameter G by sqrt [2]. The latter scaling preserves the intended dimensional relationship between conductance and the mixing term. PGE withdrawal was represented by a 10-fold increase in PDA resistance, corresponding to marked functional constriction rather than complete anatomical obliteration. These coefficients were treated as idealized model perturbations, not patient-specific pharmacodynamic estimates. Prespecified sensitivity analyses repeated the simulations with ASD resistance reductions of 30%, 50%, and 70% and PDA resistance increases of 5-, 10-, 20-, and 100-fold.

### 2.2. Computer Solver

The computer analysis was conducted using the Python computer platform (Python Software Foundation, version 3.8.3, Beaverton, OR, USA) to code all the governing Equations (1)–(11) of our TGA model. This system of equations for the blood pressure, blood flow and O_2_ saturation variables was numerically solved using the “optimize.fsolver” function from the “scipy” package (version 1.9.1) using the following parameters: tolerance for consecutive iterates xtol = 10^−4^, maximum number of function evaluations maxfev = 10^2^, and initial step bound factor = 0.1. All the runs employed in this study were checked to have a convergent iterative solution within the specified tolerance.

### 2.3. Monte Carlo Realizations

The Monte Carlo method generated 500 virtual patients using NumPy (3.5) random-number generation with a fixed seed of 20260713. Variables were sampled independently. Heart rate, SVR, PVR, ASD resistance, and PDA resistance followed lognormal distributions parameterized as X = X0 × LogNormal (0, sigma), with central internal model values of 140 beats/min, 13.0 WU·m^2^, 1.84 WU·m^2^, 7.0 WU·m^2^, and 0.18 WU·m^2^, respectively. The corresponding dispersion parameters were sigma = 0.052, 0.229, 0.291, 0.059, and 0.040. G was sampled as Binomial(5000, 0.02)/5000. No truncation was applied.

$$HR\;\sim \;140\times \mathrm{lognormal}\left(1,\;0.1\right)\;BPM$$

$$G_{asd}\;\sim \;\mathrm{binomial}\left(\mu =0.1,n=1000\right)/1000$$

$$SVR\;\sim \;13\times \mathrm{lognormal}\left(1,\;0.3\right)\;WU$$

$$PVR\;\sim \;2\times \mathrm{lognormal}\left(1,\;0.3\right)\;WU$$

$$\;R_{asd}\;\sim \;0.5\times \mathrm{lognormal}\left(1,\;0.1\right)\;WU$$

$$\;R_{pda}\;\sim \;0.18\times \mathrm{lognormal}\left(1,\;0.1\right)\;WU$$

The realized ranges were heart rate 115.6–159.8 beats/min; SVR 6.72–22.32 WU·m^2^; PVR 0.91–3.95 WU·m^2^; ASD resistance 5.98–8.93 WU·m^2^; PDA resistance 0.161–0.201 WU·m^2^; and G 0.0148–0.0256. Five hundred physiologically admissible realizations were retained; solutions with nonpositive flow or oxygen delivery or saturations outside 0–100% in any primary stage were rejected.

#### Sensitivity and Plausibility Analyses

Robustness was assessed in three ways. First, the BAS/PDA coefficients were varied factorially: ASD resistance was reduced by 30%, 50%, or 70%, with G scaled by sqrt [1/(1 − reduction)], and PDA resistance was increased 5-, 10-, 20-, or 100-fold. Second, vascular effects of PGE withdrawal were explored by increasing PVR and/or SVR by 10% or 20% in the PGE-off state. Third, the signed pressure-gradient equation for ductal flow was evaluated in the primary cohort and in a stress, grid spanning PVR 0.5–15 WU m^2^ and SVR 5–25 WU·m^2^. Baseline outputs were compared descriptively with published neonatal and TGA hemodynamic data; this was considered a physiological plausibility assessment rather than external validation.

### 2.4. Key Observables and Statistical Tests

The main objective of the study was to quantify the effect of PVR and SVR on arterial oxygen saturation, the systemic flow, the effective pulmonary flow, and the oxygen delivery. Since different patients start at different baseline values for these 4 metrics of flow and oxygenation, we consider the following normalized counterparts:

(a) Change in systemic arterial oxygen saturation (ΔS_sa_), defined as systemic arterial saturation in the PGE-off post-BAS state minus systemic arterial saturation in the PGE-on post-BAS state;
(b) Relative change in systemic blood flow (ΔQ_s_/Q_s_), defined as systemic flow in the PGE-off post-BAS state minus systemic flow in the PGE-on post-BAS state, divided by the latter;
(c) Relative change in effective pulmonary blood flow (ΔQ_p,eff_/Q_p,eff_), defined as effective pulmonary flow in the PGE-off post-BAS state minus effective pulmonary flow in the PGE-on post-BAS state, divided by the latter;
(d) Relative change in oxygen delivery (ΔDO_2_/DO_2_), defined as oxygen delivery in the PGE-off post-BAS state minus oxygen delivery in the PGE-on post-BAS state, divided by the latter.

The effects of PVR and SVR on the four outcomes were assessed using multivariable ordinary least-squares regression. Secondary comparisons retained the high-PVR and low-PVR groups defined by the upper and lower cohort tertiles. Sensitivity results are summarized as medians and interquartile ranges. Statistical significance was defined as *p* < 0.05. Because the sensitivity analyses were designed to evaluate robustness rather than estimate clinical treatment effects, emphasis was placed on direction and magnitude rather than multiplicity-adjusted hypothesis testing.

## 3. Results

The analysis included 500 virtual patients sampled using the Monte Carlo method as explained above. The hemodynamic characteristics of this cohort are shown in Table 1 for the three steps associated with the administration of PGE at birth, the performance of BAS, and the eventual discontinuation of the administration of PGE.

### 3.1. Physiological Plausibility Assessment

The PGE-on pre-BAS cohort produced a median systemic arterial saturation of 64.7% (IQR, 59.1–68.8%), consistent with the severe hypoxemia expected when restrictive interatrial communication prompts BAS [2,3,4,5]. The median heart rate was 140.8 beats/min, systemic flow was 1.86 L/min/m^2^, and pulmonary flow was 3.11 L/min/m^2^. Published electrical-cardiometry data in stable term neonates report heart rates near 126–131 beats/min and cardiac indices around 2.5–2.6 L/min/m^2^, although those values do not directly represent either parallel circuit in TGA [8]. In infants younger than 2 months with TGA and an intact ventricular septum, angiographic pulmonary blood flow has been reported at 1.3–4.8 L/min/m^2^ (mean, 3.0 L/min/m^2^), encompassing the model median [9] (Table 2). These comparisons support physiological plausibility but do not constitute patient-level external validation.

### 3.2. Effect of Baseline PVR and SVR

**Figure 1:**
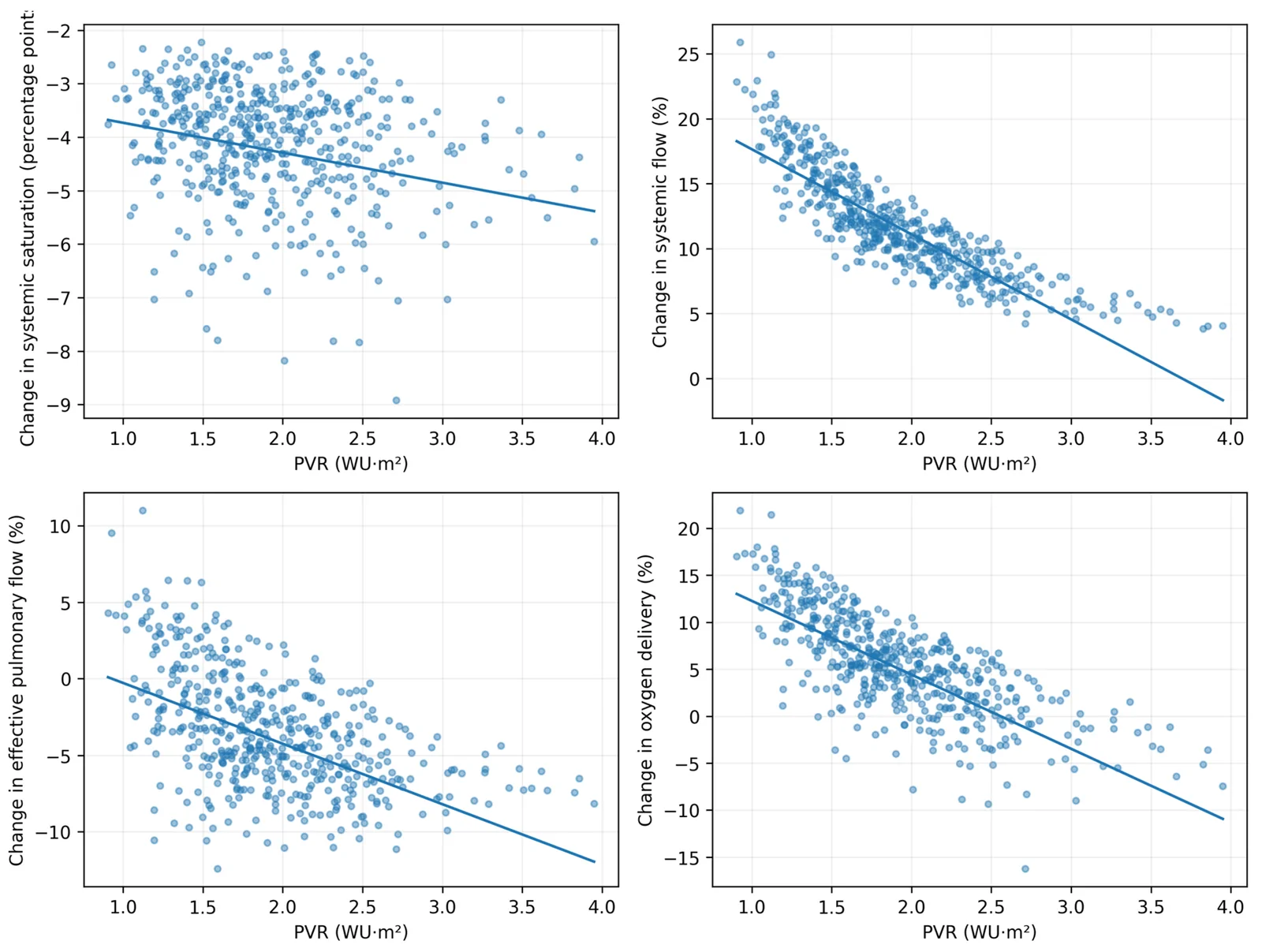
Associations between baseline PVR and the changes in systemic arterial saturation, systemic flow, effective pulmonary flow, and systemic oxygen delivery after PGE withdrawal. Changes are calculated from the PGE-on post-BAS state to the PGE-off post-BAS state. PVR = pulmonary vascular resistance; Ssa = systemic arterial oxygen saturation; Qs = systemic flow; Qp,eff = effective pulmonary flow; DO_2_ = systemic oxygen delivery.

We tested the effects of baseline PVR and SVR on the physiological response to PGE withdrawal after BAS, comparing the PGE-off post-BAS state with the PGE-on post-BAS state. The regression results are presented in Table 3 and illustrated in Figure 1. Each 1-WU·m^2^ increase in PVR was associated with a 0.560-percentage-point greater decrease in ΔSsa, a 6.556% smaller increase in ΔQs/Qs, a 3.956% greater decrease in ΔQp,eff/Qp,eff, and a 7.868% smaller increase in ΔDO_2_/DO_2_. Each 1-WU·m^2^ increase in SVR was associated with effects in the opposite direction: a 0.329-percentage-point smaller decrease in ΔSsa, a 0.518% greater increase in ΔQs/Qs, a 1.043% greater change in ΔQp,eff/Qp,eff, and a 1.113% greater increase in ΔDO_2_/DO_2_ (all *p* < 0.01).

**Figure 2:**
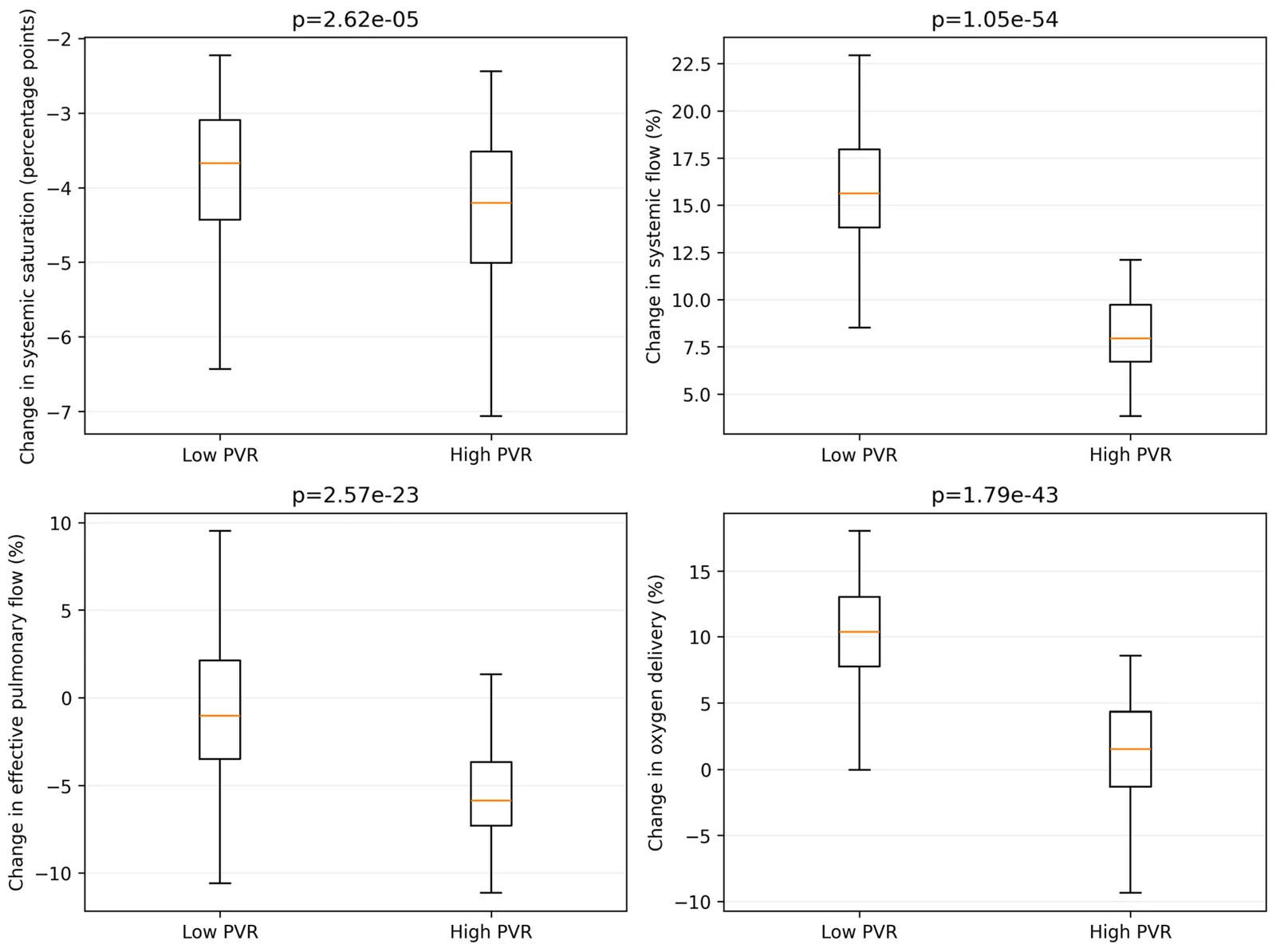
Changes in systemic arterial saturation, systemic flow, effective pulmonary flow, and systemic oxygen delivery after PGE withdrawal in the low- and high-PVR tertiles. Changes are calculated from the PGE-on post-BAS state to the PGE-off post-BAS state. *p*-values are from Mann–Whitney U tests.

The secondary analysis comparing the low- and high-PVR tertiles is displayed in Figure 2. After PGE withdrawal, the median decrease in systemic arterial saturation was −3.67 percentage points in the low-PVR group and −4.20 percentage points in the high-PVR group (*p* < 0.01). Systemic flow increased by 15.6% versus 7.9%, effective pulmonary flow changed by −1.0% versus −5.9%, and oxygen delivery increased by 10.4% versus 1.5% in the low- versus high-PVR groups, respectively (all *p* < 0.01).

### 3.3. Sensitivity Analyses

Across ASD resistance reductions of 30–70%, increasing PDA resistance 5-, 10-, and 20-fold produced progressively larger decreases in saturation (−1.75, −3.93, and −8.31 percentage points) and increases in systemic flow (4.7–6.4%, 10.0–13.3%, and 18.7–24.3%, respectively) relative to the corresponding PGE-on post-BAS state. Median oxygen delivery increased by 2.1–4.1%, 3.7–7.6%, and 4.6–10.9% (Table 4). In contrast, at a 100-fold increase in PDA resistance, effective pulmonary flow decreased by 42.7–46.8% and systemic oxygen delivery decreased by 27.4–40.8%. This reversal indicates that moderate ductal constriction and near-complete ductal closure are physiologically distinct states.

When vascular effects were added to the 10-fold PDA-resistance condition, a 10–20% PVR increase produced a larger fall in saturation (−5.47 to −7.07 percentage points) and effective pulmonary flow (−5.89% to −8.00%) than ductal constriction alone. A 10–20% SVR increase progressively reduced the gain in systemic flow and oxygen delivery; at a 20% SVR increase, median oxygen delivery decreased by 2.16%. When PVR and SVR increased together, the median oxygen-delivery change was 2.99% with 10% increases and 0.61% with 20% increases. These results show that modeling PGE solely through ductal resistance can bias the predicted magnitude and, in some combinations, the direction of the response [10].

The ductal flow equation was evaluated as a signed pressure-gradient relationship. No reverse gradient occurred in any of the 500 primary PGE-on or PGE-off simulations. In the expanded stress grid, reverse gradients occurred in 624 of 3000 combinations and first appeared at PVR approximately 3.94 WU·m^2^ under sufficiently low SVR. Therefore, the primary cohort remained in the systemic-to-pulmonary net-flow regime, but the oxygen-mixing equations should not be extrapolated to severe pulmonary hypertension with pulmonary-to-systemic ductal flow.

## 4. Discussion

The analyses confirm that PVR modulates the hemodynamic response to PGE withdrawal after BAS. Higher PVR was associated with smaller improvements in systemic flow, effective pulmonary flow, and oxygen delivery. Sensitivity analyses showed that the principal findings remained consistent when ASD resistance was reduced by 30–70% and PDA resistance was increased 5- to 20-fold. However, when PDA resistance was increased 100-fold to simulate near-complete ductal closure, effective pulmonary flow and oxygen delivery decreased. This indicates that the physiological effects of near-complete ductal closure differ from those of the primary 10-fold ductal-constriction model. These findings indicate that moderate ductal constriction and near-complete ductal closure should not be considered physiologically equivalent.

The model also demonstrates that PGE withdrawal can lower systemic arterial saturation while increasing systemic oxygen delivery. In the primary cohort, median systemic arterial saturation decreased from 74.7% after BAS with PGE to 70.9% after PGE withdrawal, while median systemic flow increased from 1.83 to 2.04 L/min/m^2^ and median systemic oxygen delivery increased from 271.4 to 283.9 mL O_2_/min/m^2^. This occurs because reduced systemic-to-pulmonary ductal runoff increases Qs sufficiently to offset the decrease in arterial oxygen content. Therefore, systemic arterial saturation alone may not accurately reflect changes in systemic oxygen delivery after PGE withdrawal. Nevertheless, these findings should not be interpreted as evidence that any decrease in saturation is clinically acceptable; saturation should be interpreted together with systemic flow and other markers of systemic perfusion and tissue oxygenation. The distinction between calculated oxygen delivery and tissue oxygen status has also been emphasized in theoretical analyses of complete-mixing circulations [11].

Another insight provided by the model is that when the PVR is low, the transpulmonary pressure gradient is low as well, which leads to a relatively high pulmonary venous pressure. Hence, after the BAS is performed, high pulmonary venous pressure induces flow through the ASD which augments the preload to the systemic ventricle [1]. As a result, the BAS is more successful in patients with low PVR as both mixing and systemic flow are improved leading to an augmented oxygen delivery.

These observations support the mechanistic hypothesis that lower PVR favors successful PGE withdrawal after BAS, but they do not establish a clinical threshold for discontinuing or restarting PGE. The model does not incorporate regional oxygen extraction, cerebral autoregulation, ventricular dysfunction, acidosis, or dynamic transitional physiology. In practice, a change in saturation should be interpreted together with systemic venous saturation, lactate, acid–base status, blood pressure, urine output, near-infrared spectroscopy, ventricular function, and the trajectory of end-organ perfusion. No absolute DO_2_ threshold for restarting PGE can be derived from these simulations.

## 5. Correlation with Clinical Observations

This model complements rather than duplicates prior computational studies of TGA, including those of Tu and Peskin and Sato et al. [8,9]. Tu and Peskin examined pulsatile pressures, flows, oxygen concentrations, and bidirectional shunting under varying defect conductance and pulmonary vascular conductance. Sato et al. used a pulsatile, time-dependent model to examine ASD, PDA, and VSD size, atrial-flow waveforms, and broad treatment strategies. The present study focuses specifically on the sequential clinical states of PGE-on pre-BAS, PGE-on post-BAS, and PGE-off post-BAS; quantifies the modifying effects of PVR and SVR across a Monte Carlo population; separates total from effective pulmonary flow; and examines the dissociation between saturation and systemic oxygen delivery during PGE withdrawal. The coefficient and vascular-effect sensitivity analyses further define the parameter ranges over which these conclusions remain valid.

A previous study tested the hypothesis that, after PDA closure, an unrestrictive interatrial communication would provide adequate circulatory mixing and prevent hypoxemia [2]. Approximately two-thirds of patients nevertheless continued to require PGE after BAS. This finding is consistent with the 60% PGE reinitiation rate reported by Pacharapakornpong et al. and the 64% reinitiation rate reported by Finan et al. among patients in whom PGE was discontinued early after BAS [3,4]. Elevated PVR has previously been proposed as a mechanism of hypoxemia after PGE withdrawal [3,4,5]. Although 12–17% of patients with TGA have been reported to show echocardiographic evidence of pulmonary hypertension [12,13], quantitative measurements of PVR in this specific clinical setting remain limited. Our model suggests that systemic oxygenation after PGE withdrawal is strongly influenced by the interaction between ductal constriction and baseline PVR, underscoring the need to interpret the effectiveness of BAS in the context of pulmonary vascular tone.

## 6. Limitations

This study has several limitations. First, the conclusions are derived from simulation and have not been externally validated against an independent cohort with simultaneous measurements of pressures, flows, vascular resistances, and oxygen saturations. The literature comparison therefore supports physiological plausibility but does not constitute external validation. Second, the ASD resistance and atrial mixing parameters used for the restrictive pre-BAS state were selected to represent the clinically expected degree of hypoxemia and were not fitted to patient-level measurements; they are therefore mechanistic representations rather than directly measured anatomical values. Third, BAS and PGE withdrawal were represented by idealized changes in resistance and mixing. Sensitivity analyses demonstrated that the principal findings were robust across 30–70% reductions in ASD resistance and 5-to-20-fold increases in PDA resistance; however, the 100-fold condition produced a reversal in the oxygen-delivery response, and the primary 10-fold model should not be interpreted as complete anatomical ductal closure. Fourth, the ventricular pressure–volume relation is linearized and uses end-diastolic and end-systolic compliance values rather than a fully time-resolved elastance waveform. Fifth, the primary oxygen-transport equations apply to net systemic-to-pulmonary ductal flow. No flow reversal occurred in the primary cohort, but reversal was observed in an expanded high-PVR stress grid; therefore, the model should not be extrapolated to severe pulmonary hypertension with pulmonary-to-systemic ductal flow without reformulating oxygen transport at the ductal junction. Sixth, unlike the time-dependent model of Sato et al. [14], the present model represents steady-state averages and applies only to TGA with an intact ventricular septum. Finally, the sampled variables were independent and therefore do not reproduce potential clinical correlations among vascular resistance, ventricular performance, hemoglobin concentration, oxygen consumption, and transitional age.

## 7. Educational Benefits

Our model has been made publicly available at https://pedicardiosim.netlify.app for use as an educational tool to help explain the physiology of TGA. Parameters that can be measured or controlled clinically can be put into the website, and then the equations will be solved to calculate the expected hemodynamic outcomes. Re-running the simulation with different states for the ASD and PDA allows users to estimate the effects of BAS and PGE discontinuation.

## Figures and Tables

**Table 1:** Hemodynamic characteristics of the virtual cohort over the 3 stages of PGE administration, performance of BAS and PGE discontinuation.

	PGE-on pre-BAS	PGE-on post-BAS	PGE-off post-BAS
HR (bpm)	140.8 (135.8–145.4)	140.8 (135.8–145.4)	140.8 (135.8–145.4)
SVR (WU·m^2^)	12.79 (11.11–15.09)	12.79 (11.11–15.09)	12.79 (11.11–15.09)
PVR (WU·m^2^)	1.84 (1.53–2.24)	1.84 (1.53–2.24)	1.84 (1.53–2.24)
Rasd (WU·m^2^)	6.95 (6.73–7.24)	3.47 (3.36–3.62)	3.47 (3.36–3.62)
Rpda (WU·m^2^)	0.18 (0.17–0.18)	0.18 (0.17–0.18)	1.80 (1.75–1.84)
G	0.020 (0.019–0.021)	0.028 (0.026–0.030)	0.028 (0.026–0.030)
Qs (L/min/m^2^)	1.86 (1.63–2.10)	1.83 (1.58–2.07)	2.04 (1.80–2.26)
Qp (L/min/m^2^)	3.11 (2.89–3.34)	3.30 (3.04–3.55)	3.11 (2.88–3.33)
Qp,eff (L/min/m^2^)	0.68 (0.64–0.70)	0.81 (0.78–0.84)	0.79 (0.75–0.83)
Ssa (%)	64.7 (59.1–68.8)	74.7 (70.6–77.9)	70.9 (66.0–74.4)
Ssv (%)	43.8 (40.7–45.8)	53.1 (51.1–54.8)	51.8 (49.0–53.8)
Spa (%)	87.4 (86.4–88.3)	88.6 (87.6–89.4)	87.8 (86.7–88.7)
DO_2_ (mL O_2_/min/m^2^)	236.8 (218.2–249.4)	271.4 (245.2–293.4)	283.9 (263.3–300.7)

HR = heart rate. SVR = systemic vascular resistance. PVR = pulmonary vascular resistance. Rasd= resistance across the atrial septal defect. Rpda = resistance across the patent ductus arteriosus. G = bidirectional atrial mixing parameter. Qs = systemic flow. Qp = pulmonary flow. Qp,eff = effective pulmonary flow. Ssa = systemic arterial oxygen saturation. Ssv = systemic venous oxygen saturation. Spa = pulmonary arterial oxygen saturation. DO_2_ = systemic oxygen delivery. bpm = beats per minute. WU·m^2^ = indexed Wood units. All columns display median (interquartile range).

**Table 2:** Physiological plausibility assessment.

Variable	Model PGE-on pre-BAS	Published Comparator	Interpretation
Heart rate	140.8 (135.8–145.4)	Term neonates: approximately 126–131 bpm [8]	Comparable neonatal scale
Systemic arterial saturation	64.7 (59.1–68.8)%	Restrictive atrial communication prompting BAS: commonly <60–70% [2,3,4,5]	Consistent with BAS-dependent hypoxemia
Systemic flow	1.86 (1.63–2.10) L/min/m^2^	Term neonatal cardiac index: approximately 2.5–2.6 L/min/m^2^ [8]	Not directly equivalent to total CO in parallel TGA
Pulmonary flow	3.11 (2.89–3.34) L/min/m^2^	TGA-IVS < 2 months: 1.3–4.8 L/min/m^2^; mean 3.0 [9]	Within reported TGA range

**Table 3:** Linear regressions for observable metrics of interest using PVR and SVR as independent factors.

	Coeff	Std Err	*p* Value
Change in systemic arterial saturation (percentage points)			
Intercept	−7.374	0.114	<0.01
PVR	−0.560	0.037	<0.01
SVR	0.329	0.007	<0.01
Relative change in oxygen delivery (%)			
Intercept	5.905	0.416	<0.01
PVR	−7.868	0.137	<0.01
SVR	1.113	0.025	<0.01
Relative change in systemic flow (%)			
Intercept	17.595	0.330	<0.01
PVR	−6.556	0.108	<0.01
SVR	0.518	0.020	<0.01
Relative change in effective pulmonary flow (%)			
Intercept	−9.662	0.297	<0.01
PVR	−3.956	0.097	<0.01
SVR	1.043	0.018	<0.01

SVR = systemic vascular resistance. PVR = pulmonary vascular resistance.

**Table 4:** Sensitivity analyses.

PDA Resistance	Delta Ssa (Points)	Delta Qs (%)	Delta Qp,eff (%)	Delta DO_2_ (%)
5-fold	−1.7 to −1.7	4.7 to 6.4	−1.6 to −1.2	2.1 to 4.1
10-fold	−3.9 to −3.9	10.0 to 13.3	−4.1 to −3.5	3.7 to 7.6
20-fold	−8.3 to −8.3	18.7 to 24.3	−9.5 to −9.4	4.6 to 10.9

## Data Availability

The original contributions presented in this study are included in the article. Further inquiries can be directed to the corresponding author.
